# 

**Published:** 1866-03

**Authors:** 


### INDEX TO VOLUME VIIB.

A

Atlanta Medical & Surgical Journal,...... 43
Atlanta Medical College,............. .... 45
Atlanta Medical Society,.................. 52
Atlanta—Returning Prosperity of,......... 85
American Medical Association,............ 92
Atlanta Medical College,................ 137
Anæthesia—New and Ready mode of Producing,............................... 179
Atlanta Medical College,................. 189
Address, introductory to the 8th course of Lectures in Atlanta Medical College,.... 196
Anæmia ................................. 254
Atlanta Medical College,................. 275
Anæsthesia, Strychnia in................. 317
Atlanta Medical College,................. 331
Amblyopia, Amaurosis and Extraction of Cataract,............... ............... 369
Anæmia, Transfusion in................... 372
Address to the Faculty,.................. 375
Amblyopia, Amaurosis and the............ 413
Asthma, Relief of Spasmodic............. 426
Atlanta Medical College,................. 427
Anæmia, Cerebro-Spinal—case of......... 498
Address,................................. 516
Abortion, Criminal....................... 520
Atlanta Medical Society—extract from minutes of........................101—161
Atlanta Medical Society—Proceedings of.. 247
Alcohol, as a dressing for wounds......... 419
Aneurism, Popliteal...................... 556

B

Bullet, Probe............ ................ 574
Bromides of Ammonium and of Potassium 23
Biographical Sketches of living New Xork Surgeons,............................... 141
Bibliographical,.......................... 378
Bibliographical,.......................... 476
Burns and Scalds, Starch Water—Application in.............. ..................... 136
Bibliographical,............ ............. 571

C

Children, nourishment of.................. 532
Cryptogamous, origin of Fever............ 334
Convulsions, infantile,................... 504
Cerebro-Spinal Meningitis, cases of....... 491
Cerebro-Spinal Meningitis,................ 27
Cholera at the port of New York in 186',. 33
Clinical Lectures, notes of................ 58
Clinic, case from Prof. Greene,............ 77
Cases reported by Dr. J. T. Banks,........ 97
Cholera, whether contagious.............. 320
Cerebro-Spinal Meningitis................ 126
Cholera, rapidity of the progress of...... 144
Cholera, plan for Quarantine for......... 144
Cerebro-Spinal Meningitis, witn report of cases,.... ............................ 145
Cholera. cause, symptoms and treatment of...................................... 152
Chloroform as an internal remedy,........ 171
Cerebro-Spinal Meningitis,................ 310
Cholera Infantile, report of a case of.... 349
Cryptogamous, origin of Fevers and other diseases,.............................. 385
Copaifera Officinalis,..................... 267
Chlorodyne, .............................. 336
Confederate Dead, list of.................. 381
Confederate Sick and Wounded,........... 235
Chlldren's Teeth,......................... 237
Caustic, new.......... ................... 558

D

Decidua, what becomes of it in Abortion,.. 15
Diptheria, Kreasote in .................. 49
Dislocation of both bones, fore-arm backwards—five months standing reduction of 61
Digitalis and its uses,.................... 466
Diptheritic Affections, Lime Inhalations in 182

F

Foreign Intelligence,...................... 81
Fever, Intermittent—Dr. Salisbury's discovery of causes of....................... 176
French Jurisprudence,..................... 527
Faculty of Medicine, Paris,............... 528

G

Gun shot Wounds, Elbow-joint—report of nine cases of........................... 218
Gonorrhæa, a new remedy in............. 267
Gossipium, remarks on the Therapntic effects of................................. 337
Gunshot wound of Bladder,............... 359
Glycosuria, with report of a case,......... 433

H

Hemorrhoids, Rectified Oil Amber in...... 256
Hospital Records,......................... 429
Hospitals, General—of Paris.............. 526
Horse Hair as a substitute for Wire,...... 71
Hypophosphites, Theraputic value of,..... 117
Hooping Cough,........................... 574

I

Inhaler, new for Sulph. Ether,........... . 143
Introductory Lecture, extracts from...... 212
Indiginous Medical Plants, lines on....... 299
Indiginous Medical Plants, lines on....... 408
International Medical Congress,... ....... 573

J

Journals Received,......................... 94

K

Kosso, superiority of as a remedy for Tape Worm,.................................. 88

L

Letter from Prof. Flint,.................. 526
Laryngoscope, the value of....... ... 133
Lectures, Summer course of............... 46

### M

Medical Education, .................... 82
Medical Association of Georgia,........... 91
Medical Works, valuable.................. 572
Medical Association of Georgia,.......... 142
Medical Association of Georgia,........... 186
Medical Assooiation of Georgia, extracts from the proceedings of................. 187
Medical Association of Georgia, transactions of.............................. 230
Medical Department, University Louisville, 279
Milk-Sickness, its Pathology and Treatment,................................ 289
Medical International Congress, .......... 332
Medical Science, contributions to
Monster, report of a case of.............. 478
Milk-Sickness, Pathology and Treatment of 481
Mott Memorial Library,.................... 525
Mecurial Collodion,....................... 525
Medical Formulæ, with remarks,.......... 543
Meteorological Observations,.............. 575

### N

Nitrate of Potash in Intermittent Fever,.. 471

Nitrous Oride and other Anœsthetics,..... 66

Nitrous Oride Gas as an Anœsthetic,...... 86

Notices &c.,.............................. 191

New York Academy of Medicine,.......... 335

New Books,................................ 519

### O

Ozone .................................... 80
Our Contributors.......................... 143
Ophthalmic Science, Recent Advances in.. 139
Ovaries, successful removal of both....... 260
Oxygen, treatment of diseases by......... 324
Our Journal............................... 473
Our Journal—Advertising medium........ 562

### P

Polypus, Intra-uterine.................... 540
Prize Essays.............................. 380
Placenta Previa........................... 50
Pericarditis, case of, with large effusion.. 80
Phytolacca Decandra...................... 193
Pharyngitis chronic....................... 510
Palmer—Arm and Leg..................... 93
Payments, list of......................... 95
Pulmonary Appoplexy..................... 529

### Q

Quinine, is it a partus accelerator?....... 340

### R

Rheumatic Fever, blister treatment of.... 326
Remarks.................................. 476
Review, Prof. Flint's Diseases of Respiratory Organs.............................. 562
Review, Prof. Flint's Principles and Practice of Medicine......................... 565

### S

Subnitrate of Bismuth, local applications of in Variola.......................... 46
Scabies, treatment of...................... 47
Stone in the Bladder, of large size........ 241
Stone, supra-pubic operation.............. 244
Shaffer, report of two cases............... 303
Symphmograph............................ 424
Scirrhus of Duodenum, Pylorus, etc.,..... 363
Syphilitic Inoculation, remarks on......... 461
Sexes, on the law of...................... 501
Sp der s Web as a styptic................. 512
Salutatory................................ 514
Strichnia, antidote for.................... 509

### T

Tongue, excision of....................... 68
Trichiniasis.............................. 88
Trichina Spiralis......................... 109
Trichina.................................. 184
Trichina and Trichinosis.................. 224
Trichinæ.................................. 229
Therapeutics, extremes in................. 276
Trichiniasis.............................. 279
Treatise on the Principles and Practice of Medicine, etc.,......................... 281
Treatise on the Diseases of the Sexual Organs of Women.......................... 287
Tumor of the Testis containing fœtal remains.................................. 37

### U

Uterine hemorrhage, remarks on........... 354
Uterus and both Ovaries successfully removed................................. 90
Uterine hemorrhage fatal case............ 314
Uterus, a case of retroflection of, cured... 367

### V

Variolous Inoculation and Vaccination, history of, with remarks.................... 1
Variola, effects of on fœtus in utero . 20
Variola, intra-uterine..................... 87
Vesico, Vaginal Fistula.................... 257
Vomiting, obstinate case of................ 352
Velpean M................................ 527
Valedictory Address...................... 343
Volume Seven, end of..................... 561

